# I Only Want Passionate Relationships: Are You Ready for That?

**DOI:** 10.3389/fpsyg.2021.673953

**Published:** 2021-06-14

**Authors:** Mar Joanpere, Gisela Redondo-Sama, Adriana Aubert, Ramon Flecha

**Affiliations:** ^1^Department of Business Management, Universitat Rovira i Virgili, Reus, Spain; ^2^Faculty of Social Sciences, University of Deusto, Bilbao, Spain; ^3^Department of Sociology, University of Barcelona, Barcelona, Spain

**Keywords:** communicative acts, communicative methodology, language of desire, language of ethics, passionate relationships, new alternative masculinities

## Abstract

Research shows the existence of a coercive dominant discourse that associates attraction with violence and influences the socialization processes of many girls and women. According to previous studies, the coercive dominant discourse constitutes a risk factor for gender violence, as men with violent attitudes and behaviors are socially presented as attractive and exciting while egalitarian and non-aggressive men are considered “not sexy.” Yet fewer evidences indicate that men acting from the New Alternative Masculinities (NAM) model overcome this double standard through verbal and non-verbal communicative acts, which tell that they do not choose women acting under the coercive dominant discourse for a relationship because they are not “jumping for joy” when meeting them. Drawing from communicative daily life stories conducted to men and women from diverse sociocultural backgrounds and ages, this article presents how language is used in concrete heterosexual sexual-affective relationships. The analysis resulting from the fieldwork focus on how NAM men’s communicative acts with women set conditions of desire. This article shows evidence on how communicative acts of NAM empowerment incorporate “language of desire,” taking a clear position for egalitarian and passionate relationships. Implications for gender violence prevention are presented.

## Introduction

Amy: When we met, he usually looked in my eyes and depending on the face, which I arrived with - showing hope or desire or enchant, then… he smiled.

Amy talks about a relationship with a man illustrating the impact that a communicative act has on her attitude before a date with him. The situation she describes shows the power of non-verbal communication (looking in my eyes) in social interaction, because the gestures and expressions they shared illuminate the way their “encounter” is going to occur. In this particular interaction, the man’s attitude aligns with what previous research in gender studies has defined as New Alternative Masculinities (NAM). Particularly, like the man in our example, those who embody the NAM model desire and choose girls who intensively desire to have a relationship with men like them. Consequently, the communicative acts underpinning this example are at the core of this article, which focuses on the language use in heterosexual sexual-affective relationships. It presents and discusses data from social interactions in the context of sexual-affective relationships and demonstrates the ways in which men whose attitudes are associated with the NAM model perform these communicative acts in diverse contexts, as well as some of the resulting effects upon women.

Research has analyzed the consequences of men’s communicative acts on women’s attitudes under the framework of the hegemonic model of masculinity (Gordon, 1997; Georgakopoulou, 2005). Recently, this debate deepens through speech and communicative acts to defining for example consent in sexual relationships, beyond the use of the words to prevent the coercive dominant discourse (Flecha et al., 2020). In a similar vein, different authors have explored the influence of the hegemonic masculinities in the existence of a coercive dominant discourse that associates attraction with violence. In this arena, Puigvert’s pioneer research analyzed the pattern of attraction in female adolescents (aged 13–16) toward boys with violent attitudes and behaviors or boys with non-violent behavior in European secondary schools (England, Spain, Cyprus, and Finland). The results show the existence of a coercive dominant discourse that associates attraction with violence and influences the socialization processes of many girls during their sexual-affective relationships’ awakening, which has been shown to constitute a risk factor of gender violence (Puigvert et al., 2019). Likewise, different authors focused on the role of language use in relation to attractiveness and have demonstrated how participating in hegemonic discourses of masculinity may lead to reproduce and reinforce the hegemonic model of masculinity in social interactions (Kiesling, 2005; Richardson, 2010).

In the current dialogic societies, the hegemonic model of masculinity coexists with the emergence of the New Alternative Masculinities approach (NAM) that shows different ways to understand and live masculinity. Men who combine attraction and equality and generate sexual desire among women represent these types of masculinities –NAM- and they are being more active working against gender violence together with women (Flecha et al., 2013). In this work, we argue that social interactions with NAM men may open new possibilities to create conditions of excitement when women meet them. There is little knowledge about the communicative situations as a result of the NAMs’ language use in heterosexual sexual-affective relationships that contribute to create these conditions, which may transform previous coercive dominant discourse and its consequences for women. This article addresses this question by presenting evidence based on the communicative acts of men performing under the NAM model. Moreover, it explores the effects that the use of a “language of desire” has on women.

This article has four sections. First, we present the state of the art in relation to the synergies between communicative acts, models of masculinity and consequences for women. The analysis of communicative acts performed by NAM men is relatively new, and the theoretical and empirical contributions come from diverse disciplines, enhancing understanding of the phenomenon. After contextualizing the topic, we present the methodology of the study and the research questions. Third, we discuss the findings of the communicative daily life stories, and finally proceed to the conclusions to address potential developments in this field of study for the future.

## Communicative Acts, Models of Masculinity and the Impact on Women

Communicative acts have been widely analyzed from sociolinguistic and pragmatics perspectives (Searle, 1969; Reich, 2011). In this arena, the intentionality underpinning each speech act is crucial in the understanding of the communicative role of language. Searle’s analysis of intentionality behind people’s utterances (Searle, 1969; Searle et al., 1983) is well recognized. However, the analysis of communicative acts looks not only at intentionality *per se*, but its relation to the consequences of the interaction among all the participants involved. The extent to which there are power or dialogic interactions in a communicative situation explains whether a relationship is more or less dialogic or egalitarian in very diverse daily life situations (Soler and Flecha, 2010). This includes also those encounters in heterosexual sexual-affective relationships. Actually, the dialogic approach contributes to the understanding of dynamics of change in intimate relationships. Resonating with this approach, Reich (2011) introduces the notion of cooperation as a relevant element to understand conversations between subjects. According to the author, each communicative act is a proposal aimed to obtain a cooperative response. Therefore, the intentions behind speech are actually dialogically oriented. Following these contributions, the analysis of communicative acts between NAM men and women includes both, the interactions they had and the cooperative responses in their relationships.

Major advancements on linguistics and pragmatics ground a crucial aspect of this article, which is the understanding of how women (in heterosexual relationships) react to communicative acts when performed by NAM men, and the extent to which this promotes an alternative to the hegemonic model of masculinity. Some works in sociolinguistics and pragmatics’ studies focus on women’s conversations as they reproduce the double standards and dominant traditional male gendered identities. Georgakopoulou (2005) studied the construction of girls’ erotic interests in boys and found that some girls’ discourses reproduce the hegemonic images of men. According to the author’s findings, girls’ speech acts distinguish between boys who can be considered as “feminine” and “soft” and boys who can be described as “tough” and “hard.” The findings in this case show that the hegemonic model of masculinity is strengthened and the differentiation between “soft” and “hard” boys remains in speech, reproducing the dominant traditional standards of masculinity.

Our analysis also includes some contributions from other disciplines such as gender and sexuality studies or social psychology, as they have also explored the models of masculinity and its construction through the language use in social contexts. Contributions from gender studies address elements such as media, cultural messages, and language uses linked to heterosexual women’s sexual behaviors. As in some of the previous contributions in sociolinguistics and pragmatics, this perspective goes in depth into this type of aspects to provide evidence that may explain women’s construction of sexuality. However, there is less emphasis on the socialization process influenced by the coercive dominant discourse that associates attraction with violence, appearing through social networks, TV, popular media, films, magazines, among many others. In this line, Kim et al. (2007) develop a discourse analysis of prime-time televisions programs frequently watched by teenagers. The conclusions state the persistence of a heteronormative discourse on sexual scripts, which reproduce male characters who are insistent and aggressive when looking for sex. As in Georgakopoulou (2005) and Gordon’s contributions (1997), Kim’s findings confirm the use of language linked to the dominant traditional model of masculinity.

The cultural dimension appears as relevant to the socialization process of many girls and women, influenced by the coercive dominant discourse that fosters attraction toward violence. Recent scientific literature on this topic, shows that coercive dominant discourse constitutes a fundamental risk factor of gender violence. This coercive dominant discourse influences many girls’ and women’s socialization into linking attractiveness to men with violent attitudes and behaviors. In this vein, research on risk factors related to gender violence developed from a preventive socialization of gender violence approach has identified that there is a coercive dominant discourse in which people with violent attitudes and behaviors are socially portrayed as attractive and exciting (Puigvert et al., 2019).

Some contributions from social psychology and interdisciplinary approaches are unveiling transformative results on women’s communicative acts and their sexual-affective relationships. According to this, Jackson and Cram (2003) conducted a large study of young heterosexual women’s talks about their sexual relationships. As a result, they argue how girls reject dominant traditional sexual conceptions by using several alternative messages against double standards. However, the authors also note that these discourses would be silenced should there not be the support from educators and teachers. This contribution is particularly relevant to our understanding about how discourses and meaning are developed by diversity of agents, including educational leaders addressing gender issues (Samul, 2020) or school principals responding to cultural diversity (Parthenis and Fragoulis, 2020), direct or indirectly involved in a particular situation and interaction. Furthermore, there is evidence of how in concrete regimes and contexts, schools, and educators impose the dominant hegemonic masculinity in students (Bhatty and Sundar, 2020).

Pioneer research on this topic presents crucial findings that resonate with the impact of communicative acts in sexual-affective relationships. Gómez’s (2015) contribution on young sexual and affective relationship, from an interdisciplinary perspective, highlights the relevance of socialization process in shaping heterosexual young men and women’s patterns of attraction by including language use and interaction with media, peers and family, among other agents. While this socialization process is characterized by attraction to dominant and aggressive masculinities, the author also argues that alternative sexual-affective relationships are built in dialogic spaces. In the same vein, Flecha and Puigvert (2010) contribute a new concept by describing dialogic spaces and relationships that mix both language of desire and ethics. Recent studies in the field, deepen on the impact of the language of ethics and double standards in the affective and sexual socialization. Rios-González et al. (2018) focus the analysis on the role that family environment and language have in the process of linking the language of ethics and the language of desire.

The analyses on men’s communicative acts about their sexuality or sexual-affective relationships exemplify the predominance of discourses and language uses that perpetuate the gender normativity as well as the hegemonic masculinity’s practices (Murphy et al., 1999; Kiesling, 2005; Richardson, 2010; Bowleg et al., 2015). From different perspectives, these contributions support the fact that heterosexual men’s conversations are not creating alternative discourses in relation to men’s sexual-affective experience. Therefore, it could be argued that the existing literature has not yet contributed to overcoming the double standard or attraction patterns.

Other works analyze heterosexual men’s communicative acts that pay more attention on the interconnections between men’s conversations and their sexual behavior and personality (Murphy et al., 1999; Bowleg et al., 2015). Murphy et al. (1999) set up an experimental investigation with men with high and low rates of likelihood to sexually harass (LSH) in their interactions with women. They discover relevant differences on non-verbal language performed by high and low LSH men. In the first case, men show more dominant and aggressive non-verbal language than the second group. However, the percentage does not differ when sexual non-verbal language was developed. In an exploration of Northern-American heterosexual black men discourses and conversations about safe sex, Bowleg et al. (2015) studied sexual practices and found how these men follow conventional masculinity patterns when they talk about sexuality. For instance, they used to blame women for their non-safe sexual practices and consequent HIV infection. According to the authors, there are relevant implications emerging from this analysis because it shows the need to plan gender-based interventions to prevent exclusionary and chauvinist masculine discourses.

Within the line of advancing research that provides transformative elements with regards to masculinity and communicative acts on sexual-affective relationships, De Meyer et al. (2014) observe a significant correlation between adolescents’ sexual life and supporting gender equality discourses. Thus, boys addressing egalitarian principles point to be more sexually satisfied and with a better communication with their girlfriends. Likewise, Rodríguez-Navarro et al. (2014) also unveil how egalitarian discourses and heterosexual alternative men are being supported, but they stress the importance of teachers and educators to foster this support. According to these contributions, it is critical to take advantage of successful interventions that result on more desirable new alternative masculinities and less dominant traditional masculinities. Examining the communicative acts that resonate with the NAM approach and its impact on heterosexual women seems critical to advance knowledge in creating new contexts for transformative interactions (Rodríguez-Navarro et al., 2014).

Through communicative analysis, Richardson (2010) identified a strong influence of peers’ conversations on their sexuality. While prior works had already underlined the impact of peer group on adolescent and children’s socialization processes (Reay, 2001; Skelton, 2002), Richardson takes a step further in relation to sexuality. The author identifies how heterosexual young men would reproduce hegemonic communicative acts in relation to their sexual practices in order to have the approval of their colleagues. Once again, verbal and non-verbal communication with other subjects is underpinning the reproduction of a certain model of masculinity. Additionally, the author also argues that young heterosexual men should daily face with contradictory gender conceptions, which are conditioning their sexual talk and not contributing to overcome associated traditional problems, as double standards or sexual mis-education. In this sense, Kiesling (2005) reaches similar conclusions in the analysis of men’s talks in university fraternities. The author observed how men perform discourses where heterosexuality and masculine solidarity is given priority, although there is a lack of profound sharing of conversations on sex and related issues. Consequently, Kiesling states that men’s rejection to share conversations about their intimacy is strongly connected with the traditional cultural conception of masculinity. Deepening on this issue in the current societies, at a legal, political, citizen, and scientific level there is an enormous concern about the communicative acts that promote or not the consent of sexual relations through communicative acts. This is a field of exploration with a crucial impact on the prevention of abusive relationships for girls (Flecha et al., 2020). In this vein, there are studies that deepen communicative acts in different environments such as nightlife, in order to identify the interactions that prevent or promote violent behaviors (Duque et al., 2020).

## Methodology

This article uses the Communicative Methodology in the design, development and analysis of the communicative acts. In line with the European Commission’s recognition of the positive impact of this methodology in the analysis of social inequalities (European Commission, 2011), special attention has been paid to the relevance of the results as a potential contribution for gender equality in sexual-affective relationships. Besides, relevant prior research has been taken into account, especially as regards the prevention of gender violence through the analysis of communicative acts analysis using the communicative approach (Portell and Pulido, 2012; Rodríguez-Navarro et al., 2014).

The article includes data collected through seven communicative daily life stories conducted to men and women from diverse sociocultural backgrounds and ages. Communicative daily life stories consist of conversations between the researcher and the participant, which in this case refers to NAM men and women involved in relationships free of violence. This qualitative technique has a narrative orientation, in which the researcher and the participants collaborate in a joint interpretation of the participant’s daily life story in relation to the topic discussed (García-Yeste, 2014).

Five NAM men were selected according to some of the features highlighted in the NAM approach (Flecha et al., 2013), namely being: self-confident, involved in egalitarian and passionate relationships, and standing up against gender violence. The two women selected had reported interactions with NAM men. The participants in the study shared their daily life stories in one session between 45 and 75 min each, creating a favorable environment to facilitate conversation and dialogue. The way to contact the participants to invite them to the study was basically done by email, and the conversations were recorded. The daily life stories were developed and analyzed in Spanish and Catalan languages (the mother tongues of the participants involved). The excerpts in this article have been translated into English.

With the aim of going in depth into specificities of NAM men’s communicative acts and their consequences for women, particular attention was paid to the understanding of the ways in which the language of desire – it is the capacity to raise attraction and be desired- was underpinning the verbal and non-verbal communication in the participant’s interactions. Therefore, they were invited to discuss about very specific aspects of the communicative acts that occur in their sexual-affective relationships, from a simple gesture or smile to significant speech. They also were invited to share motivations, desires and consequences of the language of desire on their attitudes and behaviors.

The ethical aspects were addressed through participants’ written consent. Personal data has been protected, aware that questions addressed personal and intimate relationships. All names have been anonymized accordingly. The study received the ethical approval of the Ethical Committee of the Community of Research on Excellence for All (CREA) with reference number 20210215.

## Findings

### “Jumping for Joy,” Conditions of Excitement

An important characteristic of Paul’s verbal communication was that when he talked about a particular situation in a relationship, the conditions to continue with the other person were clear and direct in his speech. He recognizes the impact of his words on the other person and his readiness to avoid any type of reaction that does not show excitement.

(1) Paul is talking about the shine in the eyes and the words he said to a woman with whom he had a relationship.

Paul: if you are in this mood (with no shine in your eyes), I don’t want to know anything about it.

Interviewer: how do you transmit this?

Paul: by talking, saying… “Hey, where’s the shine in your eyes? (eyes wide open) You are not jumping!!” (laughs) I see that you are not jumping, there is evidence! I see that you are not… and I am telling you this.

This excerpt shows a communicative act in which Paul sets certain conditions of excitement through his speech anticipating consequences if there is no “thrill” (“if you are in this mood… I do not want to know”) or reiterating his perception about the other’s “thrill” (“you are not jumping… I am telling you this”). He also uses non-verbal performance (laughs) showing consistency with his own argument related to need for excitement. Paul uses speech to trying to identify an emotional response in the woman through this particular interaction (“where’s the shine in your eyes? You are not jumping!”), while his face movements change accordingly (eyes wide open). Additionally, the use of the expression “not jumping” is metaphoric; this is not physically occurring but full of meaning in the way the relationship is build.

Amy explains a relationship in which the communicative acts made her react and increased motivation. We can identify in her words evidence about the consequence of a very particular non-verbal dimension of the communicative act. The following situation exemplifies how she changed her thoughts of a NAM man as a result of a look from him. In a sense, it resonates with Paul’s explanation about the conditions of excitement, although in this case, the effect results only from a performative non-verbal sign.

(2) Amy is explaining the moments before a date.

Amy: we knew each other very well… and just with a simple look of him, I understood everything, and I said by myself “buff… stop it”

Interviewer: was this something you had talked about before?

Amy: it was not a technical thing… we did not say “when I arrive I will look at you and…”

In this example, the words Amy use to explain her thought (“buff…stop it”) are very illustrative of the effects of his communicative act on her (“a simple look”). All the previous interactions in the relationship are attributing meaning to the performance of a look (“we knew each other very well”). Besides, she emphasizes this previously constructed meaning when she says, “a simple look,” reiterating there is no need for speech. The change this produces on her attitude becomes crucial to understand the impact of those interactions for building a type of relationship based on excitement. She continues explaining the conversations they had to maintain and increase a passionate relationship, thus prior interactions.

(3) Amy: We often talked about it and he said “if the relationship we want is this and you don’t act accordingly like that, it makes no sense that I have to lower my expectations of the relationship that I want, I’ll look for someone else” (laughs)… He is very self-confident, I didn’t doubt a word of what he said, his words, his look, made it clear that I would be the one regretting it! (face showing emphasis on the word regretting).

Amy shows her understanding of the consequences of not being “jumping for joy” with him through “laughing” when remembering his words (“I’ll look for someone else”). Remembering of these interactions explains how the conditions for excitement were built in the relationship, so that a *simple look* was enough to elicit meaning. She emphasizes her understanding with her face at verbalizing the possible consequences of her communicative acts (“I would be the one regretting it!”).

The following example describes the end of a relationship, showing the relevance of communicative acts in the construction of attraction to NAM men.

(4)
Philip: you keep on showing tenderness and love… but then the discourse and what I see is another thing.

Interviewer: what do you mean by saying “is another thing”?

Philip: yes, I said it, I saw there was no passion. I did not use the words but in the end, you are saying the same.

Philip strengthens the conditions of excitement concerning the perceived passion from the woman on him. In this case, the perceived “lack of passion” opposing the discourse of love he was told (“what I see is another thing”), has strong influence on his decision of breaking the relationship. He does not need words to talk about the expected passion. In this case, the man is empowered by acting according to his expected conditions of excitement.

According to these examples, a crucial element that underpins these cases of heterosexual relationships is the fact that NAM men do not only claim excitement through communicative acts, but they also create conditions of excitement that should be present in their interactions with women. As a result of these communicative acts, if women do not fulfill these conditions, these egalitarian men simply do not want to be with them.

### Language of Desire

The role of the language of desire appears in the daily life stories as a crucial aspect underpinning men’s communicative acts and the consequences for women. By doing this, the implications of using language of desire go beyond the communicative act itself as it contributes to set the conditions of excitement previously explained. Participants provided several stories and we find in their narratives the crucial role of this dimension in this kind of heterosexual relationships.

(5) Paul talks about sex in the context of a conversation about the expected motivation from a woman. He describes his attitude when interacting with her. He may not use these words with her, but he means these words:

Paul: I’m not gonna have sex with you and I don’t know until when. maybe never again, right?

Paul uses language of desire (“I’m not gonna have sex”) to explain how he performs in setting conditions of excitement in a relationship. He uses language of desire to describe his attitude. In this sense, there is evidence that demonstrates how communicative acts with NAM men enhances the creation of new contexts of interaction based on the language of desire.

(6) Paul describes a very concrete situation about how he uses the language of desire while considering the effect of his look into a woman with whom he has a relationship.

Paul: Wow… she was in roller-skates! Roller-skates are like heels, they make a very beautiful leg… and I looked at her from bottom to top, she noticed that, and I said, “You’ve been out like this?” Something that shows that you think this person is really attractive to you (…) The girl gets excited about that, *and this is crucial* (emphasis by slowing down speech)… and she said, “Oh, I’m coming from the street and it’s very hot” (smile) and I was like “Come on! You look great, you’re wonderful!”

The non-verbal dimension communicative acts which Paul highlights (“I looked at her from bottom to top”) attached to his words (“You’ve been out like this?”) transmits an attitude based on both body and verbal language that enhances the creation of excitement and desire through communicative acts. He makes clear how much aware he is to the effect of his communicative act on her (“she noticed that”). The communicative act involves the consequence and reaction of the other person involved in the communication (“she noticed that; “she said, ‘Oh, I’m coming from the street and it’s very hot”’). The woman’s reaction was also based on body and verbal language. He smiles when remembering her words about coming from the street, meaning that she blushed and was trying to hide her sexual excitement. The smile, at this very moment of the conversation, attributes language of desire to his narrative. It is relevant to notice how this very short interaction occurs in a few seconds and the final consequences for the woman and the relationship. Paul’s last communicative act (“Come on! You look great, you’re wonderful!”) demonstrates in which ways the language of desire generates a remarkable atmosphere for both in the relationship.

The examples presented to this moment concern the point of view of NAM men. However, we find the expression of similar consequences to the language of desire on the women interviewed. As defined in the section “Methodology,” these are women who had reported passionate relationships with egalitarian men. Next, evidence of this is presented by analyzing of some of the reactions, feelings, thoughts and concerns these women have.

(7) Amy talks about a relationship and a very particular word underpinning the communication.

Amy: it is about how he talked… he said “blonde” and he said this with desire and the people noticed that (…) Sometimes he said “you look so beautiful” (deep breath, emotion) and sometimes I would say “behave, we are in a public space” (excitement).

In this excerpt, the word “blonde” is not just a description about a physical characteristic. This word embraced profound consequences in terms of communicative acts as it was said with desire. Amy highlights the effect of his language of desire not only on her but also on the people around her (“the people noticed that”) as part of her own excitement. Continuing the conversation, her reaction to his praising (“you look so beautiful”) involves also other subjects (“behave, we are in a public space”) while the non-verbal emphasis on both utterances denote a language of desire dimension. This communicative act illustrates the impact on the women not only of NAM men attitude but also of other people’s reaction to these men attitude. Therefore, it is important to consider the extent to which the impact of the language of desire goes beyond the persons involved in the relationship, as evidenced in this example.

### Toward Egalitarian and Passionate Relationships

The third main finding is about the type of relationship emerging as a result of the NAM approach and the communicative acts associated to it. The interactions analyzed display passionate relationships tightly linked to equality, and show that subordination or superiority of women are both far away from the ideal of love.

The process of reflection on the communicative acts as an element that increases attractiveness of NAM men in egalitarian sexual-affective relationships requires a constant attitude. The dialogue underlying the interactions that raises motivation promotes this dimension. Also, it contributes to re-address situations that could potentially reduce desire.

(8) Paul explains his perceived difference in a women’s attitude if she is with a NAM man or a dominant traditional masculinity man and his reaction to this:

Interviewer: how do you materialize this in concrete interactions?

Paul: [by telling her that] the way you used to looked at “X” is not the way you are looking at me right now, and how I treat you is not how he treated you. If you want, I’m like, “what’s going on? Do I need to treat you as he did?” I’m not going to do it, I don’t want that.

Paul is clear about the contrast between someone who did not treat her well in the past and him. He verbally manifests his conditions of excitement linked to his egalitarian attitude and behavior toward her (“I’m not going to do it”). In his communicative act there is a clear rejection about a type of relationship based on mistreatment (“how I treat you is not how he treated you”) and his clear position in front of her (“I don’t want that”) attributes the speech act with an empowerment connotation filled with attractiveness, which does not put him below the other man.

In another daily life story, Claudia goes into details about the perceived equality in a relationship in which she is involved. She reflects upon the way by which this dimension is built between the subjects involved in the relationship and the extent to which equality can be constructed either attached or detached from motivation, passion or desire. We include below an excerpt from the conversation with her:

(9)
Claudia: Since the beginning, if I wasn’t equally motivated about being with him as he was with me, it didn’t work, it didn’t go well, and he would leave, or at least he showed he was willing to do so.

In her words Claudia clearly establishes a connection between equality and sexual attraction (“equally motivated about being with him”). Using language of desire, not only language of ethics, shows how the discourse of equality performs in the relationship. This connection is also linked to the possibility of passionate egalitarian relationships (“it didn’t work, it didn’t go well”). Again the conditions of excitement are included in the interaction, and become manifest in the construction (“since the beginning”) of egalitarian relationships.

The daily life stories have provided evidence about the impact of NAM men for the construction of egalitarian relationships. Furthermore, new insights about the communicative acts are to be obtained in further research.

## Conclusion

The evidence presented in this article demonstrate the ways in which NAM men are using communicative acts in a way that creates at least three consequences for women having sexual-affective relationships with them: conditions of excitement, use of the language of desire and egalitarian passion.

First, we have analyzed several examples of communicative acts that illuminate how NAM men within a sexual-affective relationship understand excitement. Accordingly, in their communicative acts they use either speech, body language or other non-verbal signs such as intonation or performative emotion to set these conditions on their interaction with women with whom they have or might have a relationship. At the same time, there is evidence with regards to the effect these conditions cause on the women interacting with them. Second, whereas there is a coercive dominant discourse in which men with violent attitudes and behaviors are socially portrayed as attractive and exciting, we found evidence of how NAM men’s use of the language of desire contribute to feeding attractiveness to them and to their relationships with women. Both discourse and attitude are performed together in them, thus showing that they do not subordinate themselves to either women or to dominant traditional masculinities. Instead, they stand up for love ideals by manifesting their desire with words, body language and signs included in the interaction, rather than an ethical stance. In this sense, the distinction between the language of desire and the language of ethics contributes new insights to pragmatic analyses of men and women interactions in and about their sexual-affective relationships. These analyses provide valuable information in the understanding of the communicative aspects of passionate and egalitarian relationships. Accordingly, our third finding points at the communicative clarification of the association between equality and passion, as a condition that can contribute to the construction of satisfactory mutually corresponding passionate relationships.

Overall, this article contributes new knowledge about the communicative acts of NAM men and the consequences for women. The discussion of the results fills the gap identified in the scientific literature, which to the best of our knowledge had not yet accounted for desire in the analysis of the different dimensions of communicative acts. Additionally, the consequences of these communicative acts on women have been also explored.

There are some limitations that this article does also put forward. On the one hand, the fieldwork deals with a topic that is related to intimate relationships. Therefore, the communicative daily life stories are sensitive to personal thoughts or feelings that may be further investigated by developing deeper trust with the researcher. Furthermore, the inclusion of other research techniques for data collection, such daily observations of the interactions between the subjects involved in the research, could enrich the obtained results. On the other hand, the role of dialogue underpinning the communicative acts, both speech and non-verbal performance, could also be further explored.

Research on the construction of new alternative masculinities has suggested a clear line of action in gender violence prevention. The findings in this study contribute new insights in the understanding about how the communicative acts performed by NAM, and the responses they get from the women with whom they get involved, enables egalitarian and passionate relationships. Moreover, the results illuminate the ways to overcome the coercive dominant discourse. Further research is needed to demonstrate how the everyday interactions of these NAM men with women, are actually contributing the prevention of gender violence, enhancing sexual-affective interactions free of violence and full of passion.

## Data Availability Statement

The datasets presented in this article are not readily available as they contain personal information. Requests to access the datasets should be directed to the authors at adriana.aubert@ub.edu.

## Ethics Statement

The studies involving human participants were reviewed and approved by CREA Ethical Committee. The participants provided their written informed consent to participate in this study.

## Author Contributions

AA and RF: conceptualization, investigation, and writing – review and editing. AA, RF, MJ, and GR-S: formal analysis. MJ and GR-S: writing – original draft. All authors contributed to the article and approved the submitted version.

## Conflict of Interest

The authors declare that the research was conducted in the absence of any commercial or financial relationships that could be construed as a potential conflict of interest. The handling editor declared a shared affiliation with an author MJ at the time of review.
